# Pós-narrativa: as crises da modernidade, o cuidado e a cura

**DOI:** 10.1590/0102-311XPT021424

**Published:** 2024-06-14

**Authors:** Roger Flores Ceccon

**Affiliations:** 1 Universidade Federal de Santa Catarina, Florianópolis, Brasil.

Em 2023, Byung-Chul Han, filósofo sul-coreano conhecido por análises sofisticadas das estruturas sociais do século XXI, lançou o livro *A Crise da Narração* ^1^, publicado no Brasil pela Editora Vozes, em que aponta a perda da capacidade de narrar na sociedade contemporânea. Inspirado por Walter Benjamin, o livro também se apoia em autores como Adorno, Baudelaire, Brecht, Freud, Heidegger, Lacan, Marx, Nietzsche, Platão, Proust, Sartre, Weber e outros. Entre pequenas fábulas e contos, Han agencia sua escrita a partir de campos do conhecimento que envolvem Filosofia, História, Sociologia, Psicologia e Literatura, constituindo um mosaico de conceitos e ideias fundamentais para quem busca a compreensão do mundo a partir das Ciências Humanas e Sociais.

A obra problematiza a sociedade atual saturada de excessos tecnológicos e informações fragmentadas, que empobrece a arte de ouvir e contar histórias e desvaloriza a experiência, a memória, o encontro, o diálogo e o afeto. Dividido em dez capítulos, aponta a crise da narração, que deixou de ser produzida atualmente, pois vivemos na época moderna pós-narrativa.

A época pós-narrativa está tomada pelo imediatismo da informação. Han, parafraseando Benjamin ^2^, aponta que o saber que vem de longe, próprio das narrativas, encontra atualmente menos ouvintes do que a informação sobre acontecimentos próximos. Portanto, a informação se sobrepôs à narrativa, e o olhar longo, lento e demorado, característico da contação de histórias, foi perdido. Somos tomados pela informação que não sobrevive ao instante do acontecimento, e a crise da modernidade se deve justamente ao fato do mundo estar tomado por esse imediatismo informacional. Narramos cada vez menos histórias, mesmo nas plataformas digitais. E histórias conectam pessoas e fomentam a empatia e a solidariedade.

A *experiência*, outro conceito estrutural do livro, pressupõe tradição e continuidade, tornando a vida narrável. Assim, narramos experiências e, quando elas se deterioram, quando não existe nada duradouro, permanece apenas a vida desnuda, caracterizada como sobrevivência. Nesse caso, o tempo atual, tomado pelo imediatismo, é reduzido a uma faixa estreita de coisas atuais, sem amplitude e profundidade. A compulsão pela atualização desestabiliza a vida, e o passado não produz mais qualquer efeito no presente.

Na era pós-narrativa, existimos sem história e somente ela desvela o futuro e nos dá esperança. Han critica a tecnologização das relações humanas, como no Snapchat, que incorpora a comunicação digital instantânea; nos *stories* do Instagram e Facebook, que não são narrações em sentido autêntico e não apresentam duração narrativa, constituindo-se como mera sequência de fotos; ou até mesmo nas *selfies*, que são válidas apenas para um momento pontual e específico. Assim, as redes sociais, que tomaram conta do mundo atual, estão localizadas no ponto zero da narrativa. Elas não são um meio de narração, mas de informação.

Na modernidade digital tardia, não há mais segredos, já que tudo é revelado. Carecemos de fantasia narrativa e encobrimos o vazio de sentido da vida postando, curtindo e compartilhando permanentemente. A crise não é mais “viver ou narrar”, mas “viver ou postar”. Entretanto, somente o mistério e o velamento das histórias, o véu que se tece em torno das coisas, é eloquente e narrativo. O velamento e a ocultação são essenciais à narração.

Han aponta que narrar significa vincular. Aquele que narra mergulha na vida e tece novos fios entre os acontecimentos. Com isso, novos relacionamentos se formam, nada fica isolado e tudo aparece repleto de sentido. Ainda, a memória constitui-se como prática narrativa que vincula novos acontecimentos, criando redes de relações e de realidades. Entretanto, na sociedade atual, percebemos a realidade quase que exclusivamente por meio de telas digitais, e o *smartphone* tomou uma dimensão tão grande que as relações são substituídas por *likes*, destruindo as possibilidades de produção narrativa.

Com relação à teoria como narração, Han é enfático: a crise atual também se apoderou da filosofia, pondo-lhe um fim. Não temos mais coragem para a filosofia, a teoria e a narração. Devemos estar cientes de que o pensamento é, ele próprio, uma narrativa que procede em etapas comunicacionais.

Inspirado nos ditos mágicos de Merseburg mencionados por Benjamin ^3^, o livro de Han aponta a narração como possibilidade de cura. A cura é a mão que narra, e a mão que narra libera a tensão, a congestão e o endurecimento, como em Freud ^4^, que também entende a dor como sintoma que indica um bloqueio na história de uma pessoa. A cura consiste em liberar o paciente desse bloqueio narrativo, em verbalizar o não narrável, no qual ele é curado quando se narra livremente. A narrativa, portanto, tem efeito terapêutico na medida em que atribui um lugar ao passado.

Para Isak Dinesen, todas as mágoas são suportáveis quando fazemos delas uma história, já que a fantasia é curativa. Entretanto, como o clima narrativo desapareceu, médicos e outros profissionais de saúde já não narram mais, tampouco têm tempo e paciência para escutar, pois a lógica da eficiência é incompatível com o espírito da narração.

A escuta, durante a narração, concentra-se não no conteúdo que está sendo dividido, mas principalmente na pessoa, no *quem* do outro. Não é um estado passivo, mas um fazer ativo, em que escutar inspira o outro a narrar e abre espaço de ressonância no qual o narrador se sente visto, sente que lhe escutam.

Para Han, o toque também tem poder de cura. Assim como a narração de histórias, encostar no outro cria proximidade e confiança, em que a mão que toca tem o mesmo efeito de cura que a voz que narra. Libera a tensão e elimina o medo, embora vivemos também em uma sociedade que não preza pelo toque, mesmo no âmbito dos serviços de saúde.

*A Crise da Narração* apresenta limites. O fato de não explorar autores reconhecidos internacionalmente por estudos sobre a temática do livro, como Paul Ricouer ^5^ ^,^ ^6^ e Jeanne Marie Gagnebin ^7^, restringe a compreensão epistemológica das configurações temporais que operam nas narrativas. Ainda, o caráter enxuto do ensaio, configurado em 133 páginas, impossibilita abordar conceitos-chave que poderiam ser melhor explorados, como *experiência*, *memória* e *tempo*. Entretanto, o livro é inovador ao articular com precisão e criticidade a crise de narração que vivemos, imersos em uma sociedade que valoriza a informação em detrimento da história. Podemos interpretar, ainda, que Han defende veementemente um devir-narrativo em que, entre outras possibilidades, está intrinsecamente contido o cuidado e a cura.

Por fim, podemos interpretar que as narrativas são capazes de potencializar vínculos, confiança e afeto no cuidado em saúde, especialmente na relação entre profissional-usuário, qualificando a assistência, a gestão e o controle social nos serviços do Sistema Único de Saúde (SUS), como também apontam Ceccon et al. ^8^. O livro de Han, embora não se destine exclusivamente a profissionais ou estudantes da área da saúde, pode provocar um *ethos* transformador ao trabalho, ao agir e ao pensar, que envolve a produção narrativa, o ouvir e o contar histórias, aumentando a capacidade de cuidar, fortemente inspirada nas bases da Saúde Coletiva brasileira.
